# Filgotinib decreases both vertebral body and posterolateral spine inflammation in ankylosing spondylitis: results from the TORTUGA trial

**DOI:** 10.1093/rheumatology/keab758

**Published:** 2021-10-14

**Authors:** Walter P Maksymowych, Mikkel Østergaard, Robert Landewé, William Barchuk, Ke Liu, Leen Gilles, Thijs Hendrikx, Robin Besuyen, Xenofon Baraliakos

**Affiliations:** Department of Medicine, University of Alberta, Edmonton, Alberta, Canada; Copenhagen Center for Arthritis Research, Center for Rheumatology and Spine Diseases, Center of Head and Orthopedics, Rigshospitalet, Glostrup; Department of Clinical Medicine, University of Copenhagen, Copenhagen, Denmark; Department of Rheumatology and Clinical Immunology, Amsterdam University Medical Center, Amsterdam; Department of Rheumatology, Zuyderland Medical Center, Heerlen, The Netherlands; Clinical Research, Gilead Sciences, Inc., Foster City, CA, USA; Clinical Research, Gilead Sciences, Inc., Foster City, CA, USA; Biometrics, Galapagos NV, Mechelen, Belgium; Medical Affairs; Clinical Development, Galapagos BV, Leiden, Netherlands; Rheumazentrum Ruhrgebiet Herne, Ruhr-University, Bochum, Germany

**Keywords:** AS, filgotinib, inflammation, MRI, therapeutics

## Abstract

**Objectives:**

To assess the effects of filgotinib on inflammatory and structural changes at various spinal locations, based on MRI measures in patients with active AS in the TORTUGA trial.

**Methods:**

In the TORTUGA trial, patients with AS received filgotinib 200 mg (*n* = 58) or placebo (*n* = 58) once daily for 12 weeks. In this post hoc analysis, spine MRIs were evaluated using the Canada–Denmark (CANDEN) MRI scoring system to assess changes from baseline to week 12 in total spine and subscores for inflammation, fat, erosion and new bone formation (NBF) at various anatomical locations. Correlations were assessed between CANDEN inflammation and clinical outcomes and Spondyloarthritis Research Consortium of Canada (SPARCC) MRI scores and between baseline CANDEN NBF and baseline BASFI and BASMI scores.

**Results:**

MRIs from 47 filgotinib- and 41 placebo-treated patients were evaluated. There were significantly larger reductions with filgotinib *vs* placebo in total spine inflammation score and most inflammation subscores, including posterolateral elements (costovertebral joints, transverse/spinous processes, soft tissues), facet joints and vertebral bodies. No significant differences were observed for corner or non-corner vertebral body inflammation subscores, spine fat lesion, bone erosion or NBF scores. In the filgotinib group, the change from baseline in the total inflammation score correlated positively with the SPARCC spine score. Baseline NBF scores correlated with baseline BASMI but not BASFI scores.

**Conclusions:**

Compared with placebo, filgotinib treatment was associated with significant reductions in MRI measures of spinal inflammation, including in vertebral bodies, facet joints and posterolateral elements.

**Trial registration:**

ClinicalTrials.gov (https://clinicaltrials.gov), NCT03117270.

Rheumatology key messages

- Filgotinib significantly reduced spinal inflammation in diverse spinal locations when compared with placebo.
- In particular, filgotinib reduced inflammation in the facet joints and posterolateral elements.
- Filgotinib ameliorates inflammation in spinal structures that are highly relevant to spinal function and mobility.

## Introduction

Axial SpA (axSpA) is a chronic inflammatory condition involving the axial joints and entheses that can lead to chronic pain, structural damage and disability [1, 2]. AS is considered a subset of axSpA. There is substantial overlap in the clinical definitions of classic AS (based on the modified New York criteria) and radiographic axSpA (r-axSpA; based on the Assessment of SpondyloArthritis International Society criteria), such that the two terminologies largely identify the same group of patients [3, 4]. Both AS and r-axSpA are characterized by sacroiliitis on conventional radiographs [3].

MRI is the optimal imaging modality for evaluating inflammatory changes in AS [5, 6]. Current methods for quantifying inflammation of the spine are highly discriminatory between active therapy and placebo, but focus on lesions in the vertebral bodies. It is assumed that inflammatory lesions in posterolateral elements and facet joints respond similarly to therapeutic intervention, but there are no data from placebo-controlled trials to confirm this. Moreover, inflammation at these locations may significantly affect spinal mobility and function, and the impact of new therapies should therefore also include an evaluation of inflammation in these regions. While inflammation is known to be a predictor of the development of structural lesions in patients with AS [7–9], the development of fat lesions has also been associated with structural lesion development [10–12]. Measurement of fat lesions in addition to inflammatory lesions may therefore be important when assessing the efficacy of potential AS therapies. The Canada–Denmark (CANDEN) MRI scoring system allows comprehensive semi-quantitative assessment of inflammation, fat, erosion and new bone formation (NBF; i.e. bone spurs and ankylosis) of the spine [13–16]. In contrast to other scoring systems, CANDEN MRI allows evaluation by anatomical location and includes all the spinal regions that can be affected in AS [13–15].

Treatment options for patients with AS who do not respond to NSAIDs currently comprise TNF-α inhibitors, the IL-17 inhibitors secukinumab and ixekizumab and the recently approved Janus kinase (JAK) inhibitor upadacitinib [1, 17, 18]. JAKs are central transmitters of pro- and anti-inflammatory cytokine signals in immune cells and are therefore interesting targets for immunomodulation [19]. Filgotinib, an oral JAK1 preferential inhibitor, reduced disease activity and improved symptoms in patients with active AS in the phase 2 TORTUGA trial (NCT03117270) [20]. In the TORTUGA trial, filgotinib significantly improved Spondyloarthritis Research Consortium of Canada (SPARCC) [21, 22] MRI inflammation scores (bone marrow oedema) in the vertebral bodies and SI joints compared with placebo [20]. However, the effects of JAK inhibitors, including filgotinib, on structural lesions in active AS are unknown and their impact on inflammation in the posterolateral part of the spine, e.g., the facet joints, the entheses of transverse and spinous processes and the surrounding soft tissues, has not been investigated.

The aim of this post hoc analysis was to evaluate the effects of filgotinib on spinal lesions, focussing on inflammatory and fat lesions in different anatomical locations of the spine in patients from the TORTUGA trial.

## Methods

### Study design

The design of the TORTUGA trial, a multicentre, double-blind, randomized trial, has been reported previously [20]. Briefly, 116 adults with active AS (as per the modified New York classification criteria, with sacroiliitis confirmed by central reading) and inadequate response or intolerance to two or more NSAIDs were treated with oral filgotinib 200 mg (*n* = 58) or placebo (*n* = 58) once daily for 12 weeks. Prior use of one TNF inhibitor was permitted (in up to 30% of enrolled patients). Patients were recruited at sites in seven countries: Belgium, Bulgaria, Czech Republic, Estonia, Poland, Spain and Ukraine. The study protocol was reviewed and approved by the central or individual independent ethics committee in each participating country (Supplementary Table S1, available at *Rheumatology* online). All patients provided written informed consent.

### CANDEN MRI scoring

During the TORTUGA trial, MRIs were conducted at baseline and week 12 (or at the early discontinuation visit). Semi-coronal T1-weighted and short tau inversion recovery MRI sequences were independently evaluated post hoc by two experts (blinded to time point and assigned treatment) according to the detailed anatomy-based CANDEN MRI method (www.carearthritis.com/mriportal/canden/index/) [13–15]. The CANDEN MRI scoring system provides overall scores for spine inflammation, fat, bone erosion and NBF in the cervical, thoracic and lumbar segments on sagittal slices of the spine (Supplementary Fig. S1, available at *Rheumatology* online) [15, 16]. Vertebral body lesions are assessed in each of 23 discovertebral units (DVUs), each unit defined by the area between horizontal lines drawn across the middle of the vertebral bodies of adjacent vertebrae. This area includes the intervertebral disc, vertebral endplates on each side of the disc and adjacent bone marrow. Vertebral body lesions are documented according to their presence in central and lateral sagittal slices. Lesions are also recorded in the facet joints, spinous processes and soft tissues at all 23 vertebral levels, in transverse processes at 17 levels from T1 to L5 and in the rib at 12 levels from T1 to T12. If a lesion is absent, a score of 0 is applied; if a lesion is present, a score of 1 or 2 is applied depending on the lesion type (a score of 6 is applied for corner and non-corner ankylosis). Additional scores of 1 or 2 are added for certain large lesions [16].

The CANDEN MRI spine inflammation score has a total scoring range of 0–614 and can be divided into a vertebral body subscore (range 0–464), comprising vertebral corner and non-corner lesions, and a posterior elements subscore (range 0–150), comprising lesions in the facet joints, spinous processes, soft tissues, transverse processes (only 17 levels from T1 to L5) and ribs (only 12 levels from T1 to T12). The inflammation score may also be divided into four different subscores: vertebral body corner inflammation subscore (only levels T12/L1–L5/S1; range 0–254), non-corner vertebral body/spondylodiscitis subscore (range 0–162), facet joints inflammation subscore (range 0–46) and posterolateral elements inflammation subscore (sum of lesions in ribs, transverse processes, spinous processes and thoracic posterolateral vertebral body at levels C7/T1–T11/T12 and soft tissue inflammation; range 0–152) [16].

The CANDEN MRI spine fat score has a total scoring range of 0–510 and can be divided into vertebral body and posterior element (facet joints) fat subscores [16]. The CANDEN MRI bone erosion score has a scoring range of 0–208 and comprises vertebral body and posterior element (facet joints) erosion subscores. The CANDEN MRI NBF total score ranges from 0 to 460 and comprises vertebral body and posterior element (facet joints) subscores [16].

### Outcome measures

Endpoints of this post hoc analysis included change from baseline to week 12 in CANDEN MRI total spine scores for inflammation, fat, bone erosion and NBF and also spine inflammation and fat subscores. Correlations were assessed between the change in CANDEN MRI inflammation total spine and subscores and the change in clinical outcomes and between the baseline CANDEN MRI NBF score (total, vertebral body and facet joints) and baseline functional (BASFI) and mobility (BASMI) measures.

### Statistical analyses

CANDEN MRI scores were treated as continuous variables and observed changes from baseline were evaluated using analysis of covariance with factors for treatment, baseline value and randomization stratification by prior TNF inhibitor use. Least squares mean changes from baseline and between-group differences with 95% CIs were calculated; *P*-values were nominal.

Spearman correlations were determined between the change from baseline in CANDEN MRI total spine and subregion inflammation scores and the change from baseline in the following clinical outcomes: CRP, AS Disease Activity Score, BASDAI, BASFI, BASMI, lumbar flexion, chest expansion, SPARCC MRI SI joint inflammation and SPARCC MRI spine inflammation 23-DVU score (changes in SPARCC MRI SI joint and spine inflammation scores were assessed as secondary endpoints in the TORTUGA trial [20]). Pearson correlations were determined between the baseline CANDEN MRI NBF score (total, vertebral body and facet joints) and baseline BASFI and BASMI scores.

The mean of the two reader scores was used to compare changes in total spine and regional inflammation scores between treatment groups; interreader intraclass correlation coefficients (ICCs) were calculated to assess the consistency and reliability of scoring between the two MRI readers, using the ICC 2.1 model. As prespecified, interreader discrepancies were resolved by an independent adjudicator if one reader determined a case was unreadable or if the change from baseline in CANDEN spine inflammation, fat lesion or erosion score differed between the two primary reviewers by ≥6 points in different directions (one reader detected an improvement, the other detected a worsening) or by ≥15 points in the same direction (both detected either improvement or worsening). Cut-offs for CANDEN scores triggering adjudication were based on the estimated smallest detectable change for the CANDEN total spine inflammation score derived in a previously reported placebo-controlled trial of adalimumab, in which the CANDEN score was used to evaluate treatment responses [16]. Final scores for cases requiring adjudication were calculated from the mean of the adjudicator’s score and the closest score of the two primary readers.

## Results

### Patient characteristics

MRI scans from 88 patients with an evaluable MRI at baseline and week 12 (or early termination visit) were evaluated (filgotinib, *n* = 47; placebo, *n* = 41) in this post hoc analysis. Baseline characteristics were generally similar between these patients and those from the TORTUGA trial who had been excluded from the present analysis because of missing MRI scans (Supplementary Table S2, available at *Rheumatology* online).

In patients with MRI scans, the mean duration of AS was longer in those on placebo than those on filgotinib (7.7 *vs* 5.3 years, respectively; Table  1). The mean baseline total spine inflammation score was higher in the filgotinib group than the placebo group, while the mean baseline NBF score was lower in the filgotinib group *vs* the placebo group (Table  1). In the filgotinib and placebo groups, 95.7% and 85.4%, respectively, had an NBF score of <100, 2.1% and 7.3% had a score of 100–<150 and 2.1% and 7.3% had a score of ≥150. The mean baseline vertebral body and facet joints CANDEN NBF scores, according to baseline subgroups for CANDEN total NBF score, are presented in Supplementary Table S3, available at *Rheumatology* online.

**Table 1:** Demographics and baseline characteristics for patients with an MRI scan

Characteristics	Filgotinib (*n* = 47)	Placebo (*n* = 41)	All (*N* = 88)
Age, years	40.4 (11.40)	42.0 (9.09)	41.1 (10.36)
Male, %	76.6	73.2	75.0
Duration of AS, years	5.3 (5.34)	7.7 (8.31)	6.4 (6.95)
Time since diagnosis, years	5.3 (5.38)	7.8 (8.44)	6.5 (7.04)
HLA-B27 positivity^a^, % of patients	95.3	92.1	93.8
ASDAS	4.3 (0.53)	4.2 (0.71)	4.2 (0.62)
MRI SPARCC spine (range 0–108)	20.6 (20.54)	15.6 (21.33)	18.2 (20.94)
MRI SPARCC SI joint (range 0–72)	7.9 (11.58)	4.9 (6.28)	6.5 (9.56)
MASES^b^	4.9 (2.74)	4.4 (3.01)	4.7 (2.86)
CANDEN MRI new bone formation score, % of patients			
<100	95.7	85.4	90.9
100–<150	2.1	7.3	4.5
≥150	2.1	7.3	4.5
Total CANDEN MRI spine inflammation score (range 0–614)	18.0 (21.35)	11.8 (17.05)	15.1 (19.61)
Total CANDEN MRI spine fat score (range 0–510)	15.4 (27.63)	11.9 (16.33)	13.8 (23.01)
Total CANDEN MRI spine bone erosion score (range 0–208)	0.5 (1.13)	0.3 (0.57)	0.4 (0.91)
Total CANDEN MRI new bone formation score (range 0–460)	17.7 (46.51)	38.1 (65.14)	27.2 (56.56)
CANDEN MRI new bone formation score, median (range)	0.0 (0.0, 288.0)	10.5 (0.0, 298.5)	3.0 (0.0, 298.5)
CANDEN MRI new bone formation score, interquartile range	0.0–16.0	0.0–50.0	0.0–24.0
Previous TNF inhibitor therapy, % of patients	8.5	12.2	10.2

Values presented as mean (s.d.) unless stated otherwise. ^a^Filgotinib, *n* = 43; placebo, *n* = 38; total, *N* = 81. ^b^Filgotinib, *n* = 39; placebo, *n* = 32; total, *N* = 71. MASES: Maastricht AS Enthesitis Score.

### Change in CANDEN MRI scores

**Fig. 1:**
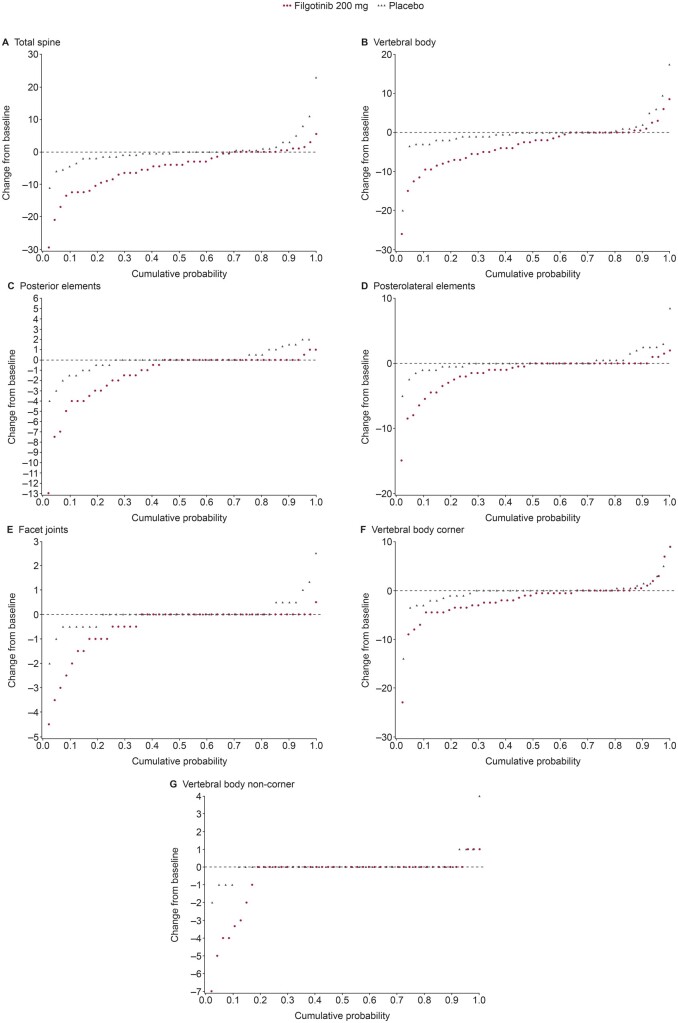
Change from baseline in CANDEN MRI score: (**A**) total spine inflammation score, (**B**) vertebral body inflammation subscore, (**C**) posterior elements inflammation subscore, (**D**) posterolateral elements inflammation subscore, (**E**) facet joints inflammation subscore, (**F**) vertebral body corner inflammation subscore, and (**G**) vertebral body non-corner inflammation subscore

**Fig. 2:**
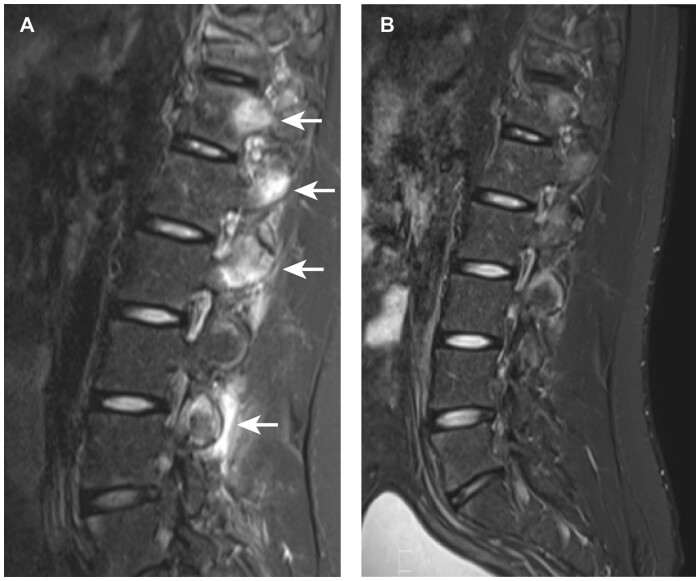
Short tau inversion recovery MRI at (**A**) baseline and (**B**) week 12 from a patient who had received filgotinib. MRI scans demonstrate posterolateral inflammation (top arrow), facet joint inflammation (middle two arrows) and soft-tissue inflammation (bottom arrow) in the lumbar spine that resolved after treatment

Total spine inflammation scores decreased from baseline in the filgotinib group but not in the placebo group (*P* < 0.001 for between-group difference; Table  2); this finding was supported by the corresponding cumulative probability plot (Fig.  1A). There were significantly greater reductions with filgotinib *vs* placebo in most spine inflammation subscores, including the posterior elements inflammation subscore (*P* = 0.006), posterolateral inflammation subscore (*P* = 0.007), vertebral body inflammation subscore (*P* = 0.009) and facet joints inflammation subscore (*P* = 0.026; Table  3). An example of reduced facet joints inflammation following treatment with filgotinib is shown in Fig.  2. No statistically significant between-group differences were observed in the change from baseline in vertebral body corner or non-corner (spondylodiscitis) inflammatory lesion subscores (Table  3). These findings were supported by cumulative probability plots (Fig.  1B–G). Total spine fat lesion scores numerically increased from baseline in the filgotinib group but decreased in the placebo group (*P* = 0.088 for between-group difference; Table  2). The between-group difference for changes in spine fat subscores did not reach statistical significance (Table  3). There were no statistically significant differences between groups for changes in total spine bone erosion (*P* = 0.20) or NBF (*P* = 0.39) scores (Table  2).

**Table 2:** Change from baseline at week 12 in CANDEN MRI total spine scores

CANDEN MRI score	Treatment group	*n*	Sample mean (s.e. )	Least squares mean (s.e.)	95% CI of treatment mean	Least squares mean of group difference (s.e. )	95% CI of group difference	Between- group *P*-value
Total spine inflammation	Filgotinib	47	−4.98 (0.96)	−4.40 (1.13)	−6.65, −2.15	−4.49 (1.21)	−6.85, −2.12	<0.001
	Placebo	41	0.29 (0.78)	0.09 (1.13)	−2.17, 2.34			
Total spine fat	Filgotinib	47	1.01 (0.62)	1.09 (0.66)	−0.22, 2.40	1.18 (0.69)	−0.18, 2.55	0.088
	Placebo	41	−0.25 (0.19)	−0.09 (0.66)	−1.40, 1.21			
Total spine bone erosion	Filgotinib	47	0.01 (0.02)	0.07 (0.03)	0.00, 0.14	0.05 (0.04)	−0.02, 0.12	0.20
	Placebo	41	−0.02 (0.03)	0.02 (0.03)	−0.04, 0.09			
Total spine new bone formation	Filgotinib	47	0.30 (0.29)	0.23 (0.31)	−0.40, 0.85	0.28 (0.34)	−0.37, 0.94	0.39
	Placebo	41	−0.01 (0.08)	−0.06 (0.31)	−0.68, 0.56			

**Table 3:** Change from baseline at week 12 in CANDEN MRI spine inflammation and spine fat subscores

CANDEN MRI subscore	Treatment group	*n*	Sample mean (s.e.)	Least squares mean (s.e.)	95% CI of treatment mean	Least squares mean of group difference (s.e.)	95% CI of group difference	Between- group *P*-value
Vertebral body inflammation^a^	Filgotinib	47	−3.43 (0.83)	−3.28 (1.02)	−5.30, −1.25	−2.84 (1.08)	−4.96, −0.73	0.009
	Placebo	41	−0.06 (0.75)	−0.43 (1.02)	−2.46, 1.59			
Posterior elements inflammation^b^	Filgotinib	47	−1.39 (0.38)	−0.88 (0.37)	−1.61, −0.14	−1.09 (0.39)	−1.85, −0.32	0.006
	Placebo	41	0.04 (0.23)	0.21 (0.37)	−0.52, 0.94			
Vertebral body corner inflammation^c^	Filgotinib	47	−1.77 (0.65)	−1.72 (0.74)	−3.18, −0.26	−1.31 (0.77)	−2.83, 0.21	0.090
	Placebo	41	−0.24 (0.47)	−0.41 (0.73)	−1.87, 1.05			
Vertebral body non-corner inflammation^d^	Filgotinib	47	−0.56 (0.23)	−0.59 (0.25)	−1.08, −0.10	−0.43 (0.26)	−0.95, 0.08	0.096
	Placebo	41	0.05 (0.13)	−0.16 (0.25)	−0.65, 0.33			
Facet joints inflammation^e^	Filgotinib	47	−0.52 (0.15)	−0.34 (0.15)	−0.62, −0.05	−0.35 (0.15)	−0.65, −0.04	0.026
	Placebo	41	0.02 (0.10)	0.00 (0.14)	−0.28, 0.29			
Posterolateral elements inflammation^f^	Filgotinib	47	−1.49 (0.44)	−0.99 (0.49)	−1.97, −0.01	−1.41 (0.52)	−2.43, −0.39	0.007
	Placebo	41	0.27 (0.29)	0.42 (0.49)	−0.55, 1.39			
Vertebral body fat^g^	Filgotinib	47	0.94 (0.61)	0.96 (0.65)	−0.33, 2.25	1.11 (0.68)	−0.22, 2.45	0.10
	Placebo	41	−0.26 (0.18)	−0.16 (0.65)	−1.44, 1.13			
Vertebral body corner fat^h^	Filgotinib	47	0.68 (0.50)	0.70 (0.53)	−0.35, 1.74	0.70 (0.56)	−0.39, 1.79	0.20
	Placebo	41	−0.12 (0.11)	−0.01 (0.52)	−1.05, 1.03			
Vertebral body non-corner fat^i^	Filgotinib	47	0.15 (0.12)	0.33 (0.15)	0.02, 0.64	0.14 (0.16)	−0.18, 0.46	0.39
	Placebo	41	0.02 (0.11)	0.19 (0.15)	−0.11, 0.50			

Data for each parameter were adjusted for corresponding baseline values. ^a^Vertebral body inflammation: increased signal in bone marrow on STIR/T2FS in a vertebral body. ^b^Posterior elements inflammation: inflammatory lesions involving the posterior elements of spine, not the vertebral bodies: facet joints, transverse processes, ribs, spinous processes and soft tissue. ^c^Vertebral body corner inflammatory lesion: inflammatory lesion at the vertebral corner (anterior and/or posterior) in at least one central slice (only at levels T12/L1–L5/S1). ^d^Non-corner inflammatory lesion (spondylodiscitis): inflammatory lesion adjacent to the endplate in any central sagittal slice but not involving the anterior or posterior vertebral corner. ^e^Facet joints inflammation: increased signal in bone marrow on STIR/T2FS scan in at least one facet of a facet joint. ^f^Posterolateral elements inflammation: sum of inflammatory lesions in the ribs, transverse and spinous processes, soft tissues and posterolateral vertebral body lesions (posterolateral vertebral body only at levels C7/T1–T11/T12). ^g^Vertebral body fat: increased signal in bone marrow on T1W scan in a vertebral body. ^h^Vertebral body corner fat: fat lesion at the vertebral comer in at least one central or lateral sagittal slice. If the original vertebral corner is distorted because of syndesmophyte formation, a fat lesion may still be scored as a corner lesion. ^i^Vertebral body non-corner fat: fat lesion adjacent to the endplate in any central sagittal slice but not involving the anterior or posterior vertebral corner. STIR: short tau inversion recovery; T1W: T1-weighted; T2FS: axial fat-saturated T2-weighted imaging.

### Interreader reproducibility

Interreader reproducibility data indicated strong agreement between the two readers for CANDEN MRI scores at baseline, with ICC values >0.50 in 12 of the 14 scores assessed, 7 of which were >0.75 (Table  4). For the change from baseline to week 12, ICC values >0.50 and >0.75 were recorded for 5 and 1 of the 14 scores, respectively (Table  4).

**Table 4:** Interreader ICCs for structural lesions

Scores	ICC	95% CI	Reader 1, mean (s.d.)	Reader 2, mean (s.d.)	All, mean (s.d.)
Baseline					
Total spine inflammation	0.833	0.784, 0.871	12.2 (15.4)	13.2 (18.9)	12.7 (17.2)
Vertebral body inflammation subscore	0.849	0.805, 0.885	9.5 (12.4)	9.8 (14.3)	9.7 (13.4)
Posterior elements inflammation subscore	0.738	0.665, 0.797	2.7 (4.6)	3.4 (5.8)	3.1 (5.2)
Vertebral body corner inflammation subscore	0.912	0.885, 0.933	5.1 (6.8)	5.3 (7.4)	5.2 (7.1)
Vertebral body non-corner inflammation subscore	0.719	0.643, 0.781	1.4 (3.2)	1.5 (3.5)	1.4 (3.3)
Facet joints inflammation subscore	0.612	0.515, 0.693	1.0 (1.8)	1.0 (2.0)	1.0 (1.9)
Posterolateral elements inflammation subscore	0.710	0.627, 0.776	3.2 (5.3)	4.6 (8.3)	4.0 (7.0)
Total spine fat	0.953	0.938, 0.964	15.5 (26.4)	15.9 (25.6)	15.7 (26.0)
Vertebral body fat subscore	0.953	0.939, 0.965	14.4 (25.6)	15.3 (25.0)	14.9 (25.3)
Posterior elements fat subscore	0.316	0.184, 0.436	1.3 (2.3)	0.5 (1.4)	0.8 (1.9)
Total spine bone erosion	0.742	0.672, 0.8	0.5 (1.6)	0.5 (1.4)	0.5 (1.5)
Vertebral body erosion subscore	0.744	0.673, 0.801	0.5 (1.6)	0.5 (1.4)	0.5 (1.5)
Posterior elements erosion subscore^a^	0	−0.141, 0.141	0.0 (0.1)	0 (0)	0.0 (0.1)
Total spine new bone formation	0.907	0.875, 0.931	28.6 (57.1)	34.5 (64.2)	31.6 (60.8)
Vertebral body new bone formation subscore	0.901	0.867, 0.927	25.3 (50.1)	31.0 (58.1)	28.1 (54.3)
Posterior elements new bone formation subscore	0.785	0.724, 0.834	3.3 (8.6)	3.5 (7.7)	3.4 (8.1)
Change from baseline to week 12					
Total spine inflammation	0.473	0.293, 0.621	−2.2 (6.7)	−3.8 (8.4)	−3.0 (7.6)
Vertebral body inflammation subscore	0.549	0.384, 0.681	−1.8 (5.9)	−2.5 (6.0)	−2.2 (5.9)
Posterior elements inflammation subscore	0.255	0.055, 0.438	−0.4 (1.7)	−1.3 (3.4)	−0.8 (2.7)
Vertebral body corner inflammation subscore	0.546	0.38, 0.678	−1.0 (3.5)	−1.4 (3.2)	−1.2 (3.4)
Vertebral body non-corner inflammation subscore	0.601	0.447, 0.721	−0.3 (1.8)	−0.3 (1.9)	−0.3 (1.9)
Facet joints inflammation subscore	0.388	0.197, 0.552	−0.2 (1.0)	−0.5 (1.3)	−0.3 (1.2)
Posterolateral elements inflammation subscore	0.216	0.015, 0.403	−0.4 (1.7)	−1.4 (3.8)	−0.9 (2.9)
Total spine fat	0.485	0.305, 0.632	0.6 (2.7)	0.5 (3.1)	0.6 (2.9)
Vertebral body fat subscore	0.488	0.308, 0.634	0.6 (2.7)	0.4 (3.1)	0.5 (2.9)
Posterior elements fat subscore	0.105	−0.11, 0.311	0.0 (0.3)	0.0 (0.3)	0.0 (0.3)
Total spine bone erosion	0.011	−0.192, 0.216	−0.1 (0.4)	0.0 (0.1)	0.0 (0.3)
Vertebral body erosion subscore	0.010	−0.196, 0.216	−0.1 (0.4)	0.0 (0.1)	0.0 (0.3)
Posterior elements erosion subscore^a^	0	−0.211, 0.211	0.0 (0.1)	0 (0)	0.0 (0.1)
Total spine new bone formation	0.518	0.344, 0.657	0.4 (2.7)	0.6 (7.0)	0.5 (5.3)
Vertebral body new bone formation subscore	0.536	0.366, 0.671	0.4 (2.7)	0.6 (6.6)	0.5 (5.0)
Posterior elements new bone formation subscore^b^	0	−0.212, 0.212	0 (0)	0.0 (0.4)	0.0 (0.3)

a All posterior elements erosion scores were 0 except for one case where one reader scored 1. ^b^All posterior elements new bone formation change scores were 0 except for one case where one reader gave a change score of 4.

### Correlation between the change in CANDEN MRI inflammation spine scores and change in clinical measures

In these exploratory post hoc analyses, in the filgotinib group, the change from baseline to week 12 in the CANDEN total MRI inflammation score correlated positively with the change in the SPARCC MRI spine score (*r* = 0.59, *P* < 0.001), while the change in the facet joints inflammation subscore correlated positively with the change in chest expansion (*r* = 0.31, *P* = 0.035; Supplementary Table S4, available at *Rheumatology* online). In the placebo group, the change from baseline to week 12 in the CANDEN total MRI inflammation score, facet joints subscore and posterolateral inflammation subscore each correlated positively with SPARCC MRI spine scores (*r* = 0.33, *P* = 0.035; *r* = 0.40, *P* = 0.010; and *r* = 0.37, *P* = 0.016, respectively), while the change in the facet joints subscore correlated negatively with lumbar flexion (*r*=−0.41, *P* = 0.009; Table S4). However, it should be noted that *P*-values were not corrected for multiple testing.

### Correlation between baseline CANDEN NBF scores and baseline functional and mobility measures

Baseline CANDEN MRI total NBF scores correlated positively with baseline BASMI scores (*r* = 0.37, *P* < 0.001; Supplementary Table S5, available at *Rheumatology* online). Baseline CANDEN MRI NBF facet joints and vertebral body scores also correlated positively with baseline BASMI (*r* = 0.39, *P* < 0.001 and *r* = 0.36, *P* < 0.001, respectively; Supplementary Table S5, available at *Rheumatology* online). There were no significant correlations between the baseline CANDEN MRI NBF scores (total, facet joints or vertebral body scores) and baseline BASFI scores (Supplementary Table S5, available at *Rheumatology* online).

## Discussion

Compared with placebo, filgotinib 200 mg decreased spine inflammation, including the posterior elements of the spine and facet joints. There is currently an absence of published, longitudinal drug data on posterior element inflammation affecting the facet joints and lateral structures. The effects of filgotinib on inflammation scores from different types of structures, such as synovial joints and entheses, are notable given that not all therapeutics may work equally well in all spinal regions. To our knowledge, this is the first randomized placebo-controlled trial to show the beneficial effect of a therapeutic agent on posterior element inflammation.

These data highlight the additional information obtained through the CANDEN MRI inflammation score compared with the more established scoring systems such as the AS spine MRI (ASspiMRI) inflammation [23], SPARCC [21] and Berlin methods [24], which do not incorporate assessments at different anatomical locations. As such, using the CANDEN MRI score in future research could help to identify patient subgroups with different disease trajectories and allow evaluation of the relationship between different lesion types over time, as well as the impact of therapy on this relationship [16].

The results from the current analysis on vertebral body inflammation, as assessed using the CANDEN scoring system, are in accordance with findings from the SPARCC analysis from the TORTUGA trial, which have been previously reported [20]. There was a slight difference between treatment groups with regard to fat lesions. Fat lesion development is a predictor of NBF in the spine in patients treated with TNF inhibitors [10–12]. However, the pathological basis of the transition from fat to new bone is not well understood [25] and there is a current lack of longitudinal MRI data regarding disease progression, particularly in patients treated with non-TNF inhibitor therapies. Longer-term data are required to evaluate the impact of reductions in fat lesion development on the progression of disease.

In the filgotinib group, positive correlations were observed between changes in the CANDEN total MRI inflammation score and SPARCC MRI spine score. However, there was no correlation between the change in CANDEN inflammation scores and clinical parameters, such as the BASDAI and BASFI. This lack of correlation with clinical parameters has been reported in trials of TNF inhibitor agents that assessed correlations with the SPARCC spine inflammation score [26]. Moderate correlations do exist in early AS, but become less evident as disease progresses [27, 28]. This might reflect the confounding effects of concomitant degenerative and mechanical disorders of the spine and the potential for the emergence of non-inflammatory pain hypersensitivity as observed in other chronic inflammatory joint diseases [29, 30].

Baseline CANDEN MRI NBF scores (total score and facet joints and vertebral body subscores) each correlated positively with baseline BASMI, but no correlation with BASFI was observed. In a study assessing the relationship between BASMI and ASspiMRI measurements in golimumab-treated patients, Baraliakos *et al.* [31] found that, at baseline, lumbar active inflammatory ASspiMRI scores correlated with lumbar flexion and lateral lumbar flexion (each *P* < 0.01), whereas chronic structural ASspiMRI also correlated with lateral lumbar flexion (*P* = 0.04). No significant correlations were found for changes from baseline in these measures at week 14. At week 104, a weak but significant correlation between the change from baseline in cervical spine chronic structural ASspiMRI score and BASMI cervical tragus-to-wall distance component score was seen [31]. These results suggest that in clinical trial participants with established AS, MRI measures of NBF, and not inflammation, were most consistently associated with restriction of mobility [32, 33]. It has been reported that spinal mobility impairment is independently determined by clinical disease activity, MRI spinal inflammation, structural damage, enthesitis and age [33]. The effect of spinal inflammation is more relevant in early AS, while spinal structural damage has a greater impact in later stages of disease [32, 33].

The ICC data show strong agreement between MRI readers at baseline, which is a strength of the study. In comparison, ICC values for the change from baseline, particularly for structural changes, were lower. However, low ICC values for change scores may reflect that variation in structural or inflammation changes between patients was limited, especially for lesions in posterolateral locations, and as such do not necessary indicate poor reliability. As expected over a 12 week study period, minimal changes in erosion and NBF were seen. In addition to the short study duration, potential limitations include the imbalance in MRI measures at baseline and the post hoc nature of the analysis. MRIs were not available from all patients, which could also have impacted results.

In conclusion, filgotinib was associated with significant reductions *vs* placebo in MRI measures of spinal inflammation at week 12 of the TORTUGA trial using the CANDEN method. In particular, filgotinib resulted in a substantial decrease in inflammation in the posterolateral elements and facet joints. These findings need to be confirmed in larger studies and long-term effects remain to be determined.

## Supplementary Material

keab758_Supplementary_Data
