# Supplementary material for: Filgotinib decreases both vertebral body and posterolateral spine inflammation in ankylosing spondylitis: results from the TORTUGA trial
Source: Rheumatology (Oxford). 2021 Oct 14;61(6):2388–97. doi: 10.1093/rheumatology/keab758 (PMC9157176; doi:10.1093/rheumatology/keab758)
Supplement: keab758_Supplementary_Data [file keab758_supplementary_data.docx]

SUPPLEMENTAL MATERIALS

**Filgotinib decreases both vertebral body and posterolateral spine inflammation in ankylosing spondylitis: results from the TORTUGA trial**

Walter P. Maksymowych,^1^^[[1]](#footnote-1)^* Mikkel Østergaard,^2,3^ Robert Landewé,^4,5^ William Barchuk,^6^ Ke Liu,^6^ Leen Gilles,^7^ Thijs Hendrikx,^8^ Robin Besuyen^9^ and Xenofon Baraliakos^10^

^1^Department of Medicine, University of Alberta, Edmonton, Canada
^2^Copenhagen Center for Arthritis Research, Center for Rheumatology and Spine Diseases, Center of Head and Orthopedics, Rigshospitalet, Glostrup, Denmark
^3^Department of Clinical Medicine, University of Copenhagen, Copenhagen, Denmark
^4^Department of Rheumatology and Clinical Immunology, Amsterdam University Medical Center, Amsterdam, the Netherlands
^5^Department of Rheumatology, Zuyderland Medical Center, Heerlen, the Netherlands
^6^Clinical Research, Gilead Sciences, Inc., Foster City, CA, USA
^7^Biometrics, Galapagos NV, Mechelen, Belgium
^8^Medical Affairs, Galapagos BV, Leiden, Netherlands
^9^Clinical Development, Galapagos BV, Leiden, Netherlands
^10^Rheumazentrum Ruhrgebiet Herne, Ruhr-University Bochum, Germany

**Contents**

[**Table S1** Ethics committees 2](#_Toc79756410)

[**Table S2** Demographics and baseline characteristics for patients with and without MRI scans 4](#_Toc79756412)

[**Table S3** Baseline vertebral body and facet joints CANDEN new bone formation scores according to baseline subgroups for CANDEN total new bone formation score 5](#_Toc79756413)

[**Table S4** Spearman correlation coefficients for the association of changes in CANDEN MRI inflammation total score and subscores with changes in clinical measures from baseline to week 12 6](#_Toc79756414)

[**Table S5** Pearson correlation coefficients for the association of baseline CANDEN MRI new bone formation total score and subscores with baseline BASFI and BASMI scores 7](#_Toc79756415)

[**Fig. S1** Anatomical location of lesions 8](#_Toc79756416)

[**Reference** 8](#_Toc79756417)

**Table S1**

Ethics committees

| **Site** | **Ethics committees** |
| --- | --- |
| CHU Sart Tilman, University of Liège, Liège, Belgium | Comité d’Ethique Hospitalo-Facultaire Universitaire de LiègeCommissie voor Medische Ethiek |
| Erasme Hospital, Brussels, Belgium | Comité d'Ethique CUB Hôpital Erasme Commissie voor Medische Ethiek |
| VIB Center for Inflammation Research, Ghent, Belgium | Commissie voor Medische Ethiek |
| CHU Ambroise Paré, Mons, Belgium | Comité d'Ethique du CHU Ambroise Paré Commissie voor Medische Ethiek |
| UMHAT "Kaspela", EOOD, Plovdiv, Bulgaria | Ethics Committee for Multicenter Trials |
| UMHAT St. Ivan Rilski, Sofia, Bulgaria | Ethics Committee for Multicenter Trials |
| MHAT - Ruse, Ruse, Bulgaria | Ethics Committee for Multicenter Trials |
| UMHAT "Kaspela", EOOD, Plovdiv, Bulgaria | Ethics Committee for Multicenter Trials |
| Second Internal Clinic UMHAT Stara Zagora, Stara Zagora, Bulgaria. | Ethics Committee for Multicenter Trials |
| CCR Czech a.s., Pardubice, Czech Republic | Eticka komise Fakultni nemocnice Kralovske Vinohrady |
| Revmatologicka ambulance, Nusle, Czech Republic | Eticka komise Fakultni nemocnice Kralovske Vinohrady |
| University of Veterinary and Pharmaceutical Sciences Brno, Brno, Jihomoravský, Czech Republic | Eticka komise Fakultni nemocnice Kralovske Vinohrady |
| North Estonian Medical Centre, Tallinn, Estonia | Tallinn Medical Research Ethics Committee |
| East Tallinn Central Hospital, Tallinn, Estonia | Tallinn Medical Research Ethics Committee |
| Klinische Forschung Berlin, Berlin, Germany | Ethik-Kommission der medizinischen Fakultät der Albert-Ludwigs-Universität Freiburg i.Br. |
| University of Freiburg, Freiburg, Germany | Ethik-Kommission der medizinischen Fakultät der Albert-Ludwigs-Universität Freiburg i.Br. |
| Krankenhaus St. Josef, Wuppertal, Germany | Ethik-Kommission der medizinischen Fakultät der Albert-Ludwigs-Universität Freiburg i.Br. |
| University Hospital Mainz, Mainz, Germany | Ethik-Kommission der medizinischen Fakultät der Albert-Ludwigs-Universität Freiburg i.Br. |
| Charité-Universitätsmedizin Berlin, Berlin, Germany | Ethik-Kommission der medizinischen Fakultät der Albert-Ludwigs-Universität Freiburg i.Br. |
| Centrum Medyczne AMED, Warsaw, Poland | Komisja Bioetyczna przy WIL |
| Twoja Przychodnia Centrum Medyczne Nowa Sol, Nowa Sol, Poland | Komisja Bioetyczna przy WIL |
| ClinicMed Daniluk, Białystok, Poland | Komisja Bioetyczna przy WIL |
| Jan Biziel University Hospital No. 2, Bydgoszcz, Poland | Komisja Bioetyczna przy WIL |
| Szpital Specjalistyczny, Bytom, Poland | Komisja Bioetyczna przy WIL |
| Wroclaw Medical University, Wrocław, Poland | Komisja Bioetyczna przy WIL |
| National Institute of Geriatrics, Rheumatology and Rehabilitation, Warsaw, Poland | Komisja Bioetyczna przy WIL |
| Ai Centrum Medyczne sp. z o.o. sp.k, Poznan, Poland | Komisja Bioetyczna przy WIL |
| Universidad de Málaga, Málaga, Spain | CEIC Hospital Universitario Madrid Monteprincipe |
| Hospital Universitari Parc Taulí Sabadell, Medicine Department UAB, Barcelona, Spain | CEIC Hospital Universitario Madrid Monteprincipe |
| Hospital Clínico Universitario de Santiago, Instituto de Investigación Sanitaria de Santiago (IDIS), Santiago de Compostela, Spain | CEIC Hospital Universitario Madrid Monteprincipe |
| Hospital Fuenlabrada, Fuenlabrada, Spain | CEIC Hospital Universitario Madrid Monteprincipe |
| Hospital Universitario Virgen Macarena, Sevilla, Spain | CEIC Hospital Universitario Madrid Monteprincipe |
| Communal Establishment of Health Protection "Kharkiv City Clinical Hospital #8", Kharkiv, Ukraine | LEC Communal Institution of Healthcare Kharkiv City Clinical Hospital #8 |
| M.V.Sklifosovskyi Poltava Regional Clinical Hospital, Poltava, Ukraine | LEC Poltava Reg.Clin.Hosp.n.a.M.V.Sklifosovskogo |
| National Pirogov Memorial Medical University, Vinnytsia, Ukraine | LEC Vinnytsia M.I.Pyrogov Regional Clinical Hospital  LEC Scient.&Research Instit.of Invalid Rehabiltation of Vinnytsia M.I.Pyrogov Nat.Med.Univer. |
| Medical Clinical Investigational Center of Medical Center Health Clinic LLC, Vinnytsia, Ukraine | LEC Med.Cl.Invest.Center Health Clinic |
| SI NSC M.D. Strazhesko Institute of Cardiology, NAMS of Ukraine, Kiev, Ukraine | LEC SI National Scientific Center Acad. M.D.Strazhesko |
| Danylo Halytsky Lviv National Medical University, Lviv, Ukraine | LEC CH of State Border Service of Ukraine (Military Base 2522) |
| Railway Clinical Hospital of Uzhorod Station of Lviv Railroad Administration, Uzhorod, Ukraine | LEC Zakarpatian Regional Clin.Hosp. n.a.A.Novak |
| Consult-Diagnostic Center of Pecherskyi District of Kyiv, Kyiv, Ukraine | LEC CNCE Consult-Diagnostic Center of Pecherskyi District of Kyiv |
| I. Horbachevsky Ternopil National Medical University, Ternopil, Ukraine | LEC Ternopil Regional Council Ternopil University Hospital |

**Table S2**Demographics and baseline characteristics for patients with and without MRI scans

| **Characteristic** | **Evaluable patients  with MRI scans** | | **Excluded patients  without MRI scans** | | **Total population** | |
| --- | --- | --- | --- | --- | --- | --- |
|  | n | Mean (SD) | n | Mean (SD) | N | Mean (SD) |
| Age, years | 88 | 41.1 (10.36) | 28 | 43.5 (10.17) | 116 | 41.7 (10.32) |
| Male, % of patients | 88 | 75.0 | 28 | 71.4 | 116 | 74.1 |
| Duration of AS, years | 88 | 6.4 (6.95) | 28 | 6.8 (5.45) | 116 | 6.5 (6.60) |
| Time since diagnosis, years | 88 | 6.5 (7.04) | 28 | 7.0 (5.49) | 116 | 6.6 (6.68) |
| HLA-B27 positivity, % of patients | 81 | 93.8 | 26 | 100.0 | 107 | 95.3 |
| BASFI | 88 | 6.9 (1.57) | 28 | 7.0 (1.35) | 116 | 6.9 (1.51) |
| BASMI | 87 | 5.1 (1.66) | 27 | 5.4 (1.37) | 114 | 5.2 (1.60) |
| BASDAI | 88 | 7.1 (1.22) | 28 | 6.8 (1.31) | 116 | 7.0 (1.24) |
| ASDAS | 88 | 4.2 (0.62) | 28 | 4.1 (0.94) | 116 | 4.2 (0.71) |
| MRI SPARCC spine (range 0–108) | 88 | 18.2 (20.94) | 18 | 7.8 (10.29) | 106 | 16.5 (19.90) |
| MRI SPARCC SIJ (range 0–72) | 88 | 6.5 (9.56) | 21 | 4.7 (7.34) | 109 | 6.1 (9.17) |
| MASES | 71 | 4.7 (2.86) | 24 | 3.9 (2.73) | 95 | 4.5 (2.83) |
| Previous TNF inhibitor therapy, % of patients | 88 | 10.2 | 28 | 7.1 | 116 | 9.5 |

Data are mean (SD) unless otherwise indicated.

AS, ankylosing spondylitis; ASDAS, Ankylosing Spondylitis Disease Activity Score; BASDAI, Bath Ankylosing Spondylitis Disease Activity Index; BASFI, Bath Ankylosing Spondylitis Functional Index; BASMI, Bath Ankylosing Spondylitis Metrology Index; HLA, human leukocyte antigen; MASES, Maastricht Ankylosing Spondylitis Enthesitis Score; MRI, magnetic resonance imaging; SD, standard deviation; SIJ, sacroiliac joint; SPARCC, Spondyloarthritis Research Consortium of Canada; TNF, tumour necrosis factor.

**Table S3**Baseline vertebral body and facet joints CANDEN new bone formation scores according to baseline subgroups for CANDEN total new bone formation score

| **Treatment group** | **Total CANDEN new bone formation score at baseline** | **n** | **Mean CANDEN vertebral body new bone formation score (SE)** | **Mean CANDEN facet joint new bone formation score (SE)** |
| --- | --- | --- | --- | --- |
| Filgotinib | <100 | 45 | 8.5 (15.13) | 1.0 (3.47) |
|  | 100 to <150 | 1 | 114.0 (NA) | 3.5 (NA) |
|  | ≥150 | 1 | 261.0 (NA) | 27.0 (NA) |
|  | All new bone formation scores | 47 | 16.1 (42.29) | 1.6 (5.09) |
| Placebo | <100 | 35 | 11.7 (16.91) | 2.7 (5.75) |
|  | 100 to <150 | 3 | 125.0 (6.93) | 9.8 (3.21) |
|  | ≥150 | 3 | 190.0 (62.95) | 28.2 (10.07) |
|  | All new bone formation scores | 41 | 33.1 (57.64) | 5.1 (8.97) |

CANDEN, Canada-Denmark; NA, not applicable; SE, standard error.

**Table S4**Spearman correlation coefficients for the association of changes in CANDEN MRI inflammation total score and subscores with changes in clinical measures from baseline to week 12

|  |  | **Spearman correlation coefficients** | | | | | |
| --- | --- | --- | --- | --- | --- | --- | --- |
|  |  | **Change in CANDEN scores from baseline to week 12** | | | | | |
|  |  | Inflammation total | | Facet joints | | Posterolateral | |
|  |  | Filgotinib | Placebo | Filgotinib | Placebo | Filgotinib | Placebo |
|  |  | (n=46) | (n=41) | (n=46) | (n=41) | (n=46) | (n=41) |
| C-reactive protein | r p-value | 0.27 0.073 | −0.05 0.773 | 0.21  0.154 | 0.12 0.463 | 0.21  0.171 | 0.05 0.755 |
| ASDAS | r p-value | 0.24 0.102 | 0.09 0.567 | 0.19 0.208 | −0.05 0.743 | 0.20 0.189 | 0.03 0.838 |
| BASDAI | r p-value | 0.12 0.435 | 0.18 0.259 | 0.19 0.197 | −0.05 0.777 | 0.19 0.204 | 0.13 0.402 |
| BASFI | r p-value | −0.10 0.517 | 0.08 0.623 | 0.01 0.945 | −0.08 0.619 | −0.00 0.985 | 0.08 0.638 |
| BASMI linear | r p-value | −0.12 0.417 | −0.00 0.983 | 0.14 0.344 | 0.14 0.398 | 0.07 0.642 | 0.11 0.501 |
| Lumbar flexion | r p-value | 0.25 0.091 | −0.24  0.126 | 0.07 0.630 | −0.41 **0.009** | 0.18 0.230 | −0.25 0.119 |
| Chest expansion | r p-value | 0.16 0.278 | −0.04 0.813 | 0.31 **0.035** | −0.12 0.464 | 0.15 0.306 | −0.08 0.618 |
| SPARCC MRI SIJ | r p-value | 0.20 0.176 | 0.11 0.509 | 0.23 0.118 | 0.09 0.555 | 0.25 0.093 | 0.18 0.258 |
| SPARCC MRI spine | r p-value | 0.59 **<0.001** | 0.33 **0.035** | 0.15 0.328 | 0.40 **0.010** | 0.11  0.447 | 0.37 **0.016** |

ASDAS, Ankylosing Spondylitis Disease Activity Score; BASDAI, Bath Ankylosing Spondylitis Disease Activity Index; BASFI, Bath Ankylosing Spondylitis Functional Index; BASMI, Bath Ankylosing Spondylitis Metrology Index; CANDEN, Canada-Denmark; MRI, magnetic resonance imaging; SIJ, sacroiliac joint; SPARCC, Spondyloarthritis Research Consortium of Canada.

**Table S5**Pearson correlation coefficients for the association of baseline CANDEN MRI new bone formation total score and subscores with baseline BASFI and BASMI scores

|  | **Pearson correlation coefficients, n=87 Baseline CANDEN new bone formation** **scores** | | | |
| --- | --- | --- | --- | --- |
|  |  | New bone formation total | Facet joints | Vertebral body |
| BASFI | r | 0.09 | 0.16 | 0.08 |
|  | p-value | 0.391 | 0.127 | 0.459 |
| BASMI | r | 0.37 | 0.39 | 0.36 |
|  | p-value | <0.001 | <0.001 | <0.001 |

BASFI, Bath Ankylosing Spondylitis Functional Index; BASMI, Bath Ankylosing Spondylitis Metrology Index; CANDEN, Canada-Denmark; MRI, magnetic resonance imaging.

**Fig. S1**Anatomical location of lesions


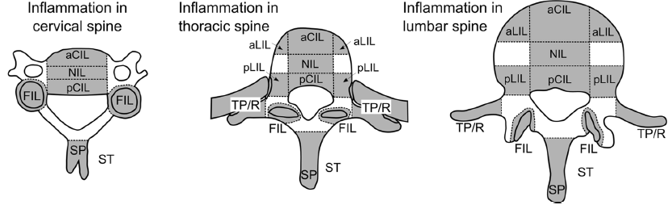


aCIL, anterior corner inflammatory lesion; aLIL, anterior lateral inflammatory lesion; FIL, facet joint inflammatory lesion; NIL, non-corner inflammatory lesion; pCIL, posterior corner inflammatory lesion; pLIL, posterolateral inflammatory lesion; SP, spinous process inflammatory lesion; ST, soft tissue inflammatory lesion; TP/R, transverse process/rib. Adapted by permission from BMJ Publishing Group Limited. Krabbe S, et al. [1]. *RMD Open* 2018;4:e000624. © 2018.

# Reference

1. Krabbe S, Sorensen IJ, Jensen B, et al. Inflammatory and structural changes in vertebral bodies and posterior elements of the spine in axial spondyloarthritis: construct validity, responsiveness and discriminatory ability of the anatomy-based CANDEN scoring system in a randomised placebo-controlled trial. *RMD Open* 2018;4:e000624.

1. * Corresponding author at [walter.maksymowych@ualberta.ca](mailto:walter.maksymowych@ualberta.ca) [↑](#footnote-ref-1)
